# Betrixaban for Deep Venous Thrombosis Prevention in a Patient with End-Stage Renal Disease on Intermittent Hemodialysis

**DOI:** 10.4236/jbm.2026.145027

**Published:** 2026-05-26

**Authors:** Victoria M. Stevens, Craig J. Furnish, Tyree H. Kiser

**Affiliations:** 1 Department of Clinical Pharmacy, University of Colorado Hospital, Aurora, CO, USA; 2 Department of Clinical Pharmacy, University of Colorado Skaggs School of Pharmacy and Pharmaceutical Sciences, Aurora, CO, USA

**Keywords:** Betrixaban, Direct-Acting Oral Anticoagulant, Venous Thromboembolism, Prophylaxis, HIT, Dialysis, End-Stage Renal Failure

## Abstract

Betrixaban is an oral factor Xa inhibitor that is indicated for venous thromboembolism (VTE) prophylaxis in hospitalized patients. It has minimal renal excretion, but it is not well studied in patients with severe renal dysfunction or patients with end-stage renal disease (ESRD). In this evaluation, we discuss betrixaban use in a patient with ESRD on intermittent hemodialysis (HD) and a history of heparin-induced thrombocytopenia (HIT) and contraindication to heparin therapy due to a positive platelet factor 4 IgG antibody. The patient was started on betrixaban for VTE prophylaxis, dosed as 80 mg once followed by 40 mg daily for a total of 15 days of therapy before they were discharged. The patient was continued on their home dialysis schedule of three times weekly and received a total of seven sessions of HD while admitted. Several anti-Xa levels collected after the second dose of betrixaban ranged between 0.12 and 0.32 IU/mL, which were within the predicted therapeutic range for betrixaban based on previous pre-clinical studies. A trough level drawn on day 7 of 0.15 IU/mL indicated the absence of accumulation. Betrixaban was well tolerated with no reported bleeding or VTE events. This case report demonstrates the successful use of betrixaban without accumulation for a patient with ESRD on HD. Betrixaban may be a potential choice when standard options are unsuitable for VTE prophylaxis or in patients with contraindications to other treatments.

## 1. Introduction

Venous thromboembolism (VTE), including deep vein thrombosis (DVT) and pulmonary embolism (PE), is a known cause of increased morbidity and mortality. Hospitalized patients are at increased risk of VTE for many reasons, including reduced mobility, infection, and recent trauma or surgery [1] [2]. As a result, the American College of Chest Physicians (ACCP) recommends the use of chemoprophylaxis for patients admitted to the hospital at high risk of VTE development [1].

Betrixaban is an oral factor Xa inhibitor that is FDA approved for VTE prophylaxis in adult patients who are hospitalized for an acute medical illness and are at risk for thromboembolic complications [3] [4]. Betrixaban is a direct oral anticoagulant (DOAC) that works by inhibiting factor Xa, thereby decreasing thrombin generation [3]. Advantages of betrixaban over other DOACs, such as apixaban and rivaroxaban, are that betrixaban requires little to no renal excretion or hepatic metabolism for clearance. Less than 1% of the drug is metabolized by hepatic enzymes, and only about 5% to 10% of the oral dose undergoes renal elimination, making it the least renally cleared of all DOACs [3] [5]–[7]. Betrixaban is primarily excreted in feces by biliary secretion (85%) [3] [5]–[7].

The APEX trial showed that, when compared to enoxaparin for the prevention of VTE in hospitalized patients, betrixaban had a trend towards benefit in the overall study population with no statistically significant difference in major bleeding rates [8]. However, this study excluded patients with creatinine clearance (CrCl) values less than 15 mL/min [8]. Here we describe a patient case of betrixaban use in a patient with end-stage renal disease (ESRD) on intermittent hemodialysis (HD). The Colorado Multiple Institutional Review Board reviewed the study, and patient consent was obtained for the additional laboratory testing and publication of the findings.

## 2. Case Report

A 61-year-old female was hospitalized in the medical intensive care unit with shortness of breath secondary to hypertensive emergency and flash pulmonary edema. Her past medical history included systemic lupus erythematosus and hemolytic uremic syndrome complicated by ESRD on intermittent HD three times weekly, outpatient. Other past medical history included hypertension, chronic obstructive pulmonary disease, and heart failure with preserved ejection fraction. The patient’s home medications are listed in Table 1. VTE prophylaxis was indicated because the patient was admitted to the intensive care unit with limited mobility and had a Padua score > 4. Of note, the patient had a listed allergy to heparin specified as thrombocytopenia. A year prior to admission, the patient had a positive heparin-induced platelet IgG antibody test (Heparin-PF4), but the serotonin-release assay (SRA) resulted as indeterminate. The patient’s baseline platelet count on this admission was <150 × 10^9^/L. Repeat Heparin-PF4 antibody and SRA tests were not available within the last 100 days prior to hospitalization. Therefore, a history of heparin-induced thrombocytopenia (HIT) and current risk of acute HIT could not be ruled out or accurately assessed, so a clinical decision was made to avoid heparin products. Rivaroxaban was avoided due to a paucity of data for VTE prophylaxis in ESRD. Fondaparinux was also contraindicated because of the patient’s history of ESRD. As such, betrixaban was chosen for VTE prophylaxis.

On the morning of hospital Day 1, the patient received 4 hours of HD. The patient was then started on betrixaban with a loading dose of 80 mg, followed by 40 mg daily starting on hospital Day 2. The usual loading and maintenance doses (e.g., 160 mg load, followed by 80 mg daily) were adjusted by 50% based on the dosing recommendations for renal impairment provided by the manufacturer [3]. The patient was not receiving any P-glycoprotein inhibitors or other medications with strong betrixaban drug interactions. Written consent was obtained from the patient to monitor anti-Xa levels. All levels were analyzed using a previously reported heparin and enoxaparin calibrated anti-Xa assay that was calibrated for DOACs, including rivaroxaban and apixaban [9] [10].

A timeline of coagulation levels can be found in Table 2 and Table 3. Baseline INR and prothrombin time (PT) were 1.1 IU/mL and 14.3 seconds, respectively, which were both normal. An anti-Xa level collected about 14 hours after the loading dose was 0.18 IU/mL. On hospital Day 2, the patient was started on betrixaban 40 mg. Anti-Xa levels collected about 1 hour (peak), 10 hours, 17.5 hours, and 25 hours (trough) after the first 40 mg dose were as follows: 0.19, 0.32, 0.19, and 0.12 IU/mL. The patient did receive HD between two of the aforementioned levels. Pre-HD anti-Xa level was 0.19 IU/mL. A level collected immediately after a 4-hour HD session was 0.12 IU/mL. Four days later, the anti-Xa level trough level was 0.15 IU/mL, and the INR was 1.1.

The patient was continued on betrixaban 40 mg daily until they were discharged from the hospital. They received a total of 15 days of therapy. During their hospital stay, they were kept on their outpatient dialysis schedule of HD three times weekly. No clinical or laboratory evidence of drug accumulation was noted. The patient did not report any adverse effects while on betrixaban. Nor was the patient noted to have any bleeding or VTE development during their hospitalization.

## 3. Discussion

For patients with renal failure, the mainstay of therapy for VTE prophylaxis is subcutaneous heparin. For patients with renal failure and HIT, there are limited therapeutic options for VTE prophylaxis. To the authors’ knowledge, this is the first case report describing the use of betrixaban for VTE prophylaxis in a patient with ESRD and a history of HIT. Several anti-Xa levels were collected and showed presence of betrixaban at expected prophylactic concentrations without continued accumulation in this patient.

From early *in vitro* studies, it was identified that betrixaban serum concentrations of 5 to 25 ng/mL produce equivalent levels of anticoagulant activity to prophylactic dosing of fondaparinux 2.5 mg subcutaneous [5] [11] [12]. To achieve these target serum levels, phase I data in healthy patients predicted that betrixaban doses of 30 to 80 mg would be needed [13]. Additionally, pre-clinical studies projected that betrixaban concentrations of 5 to 25 ng/mL would result in anti-Xa levels between 0.09 and 0.44 IU/mL [5].

In the EXPERT trial, betrixaban was compared to enoxaparin for VTE prophylaxis after total knee replacement [5]. In this trial, investigators also evaluated betrixaban concentrations and anti-Xa levels. Levels were collected on Day 2, discharge (average length of stay was 4 days), and at the mandatory follow-up venogram on Day 10 to 14. Average betrixaban concentrations at a dose of 30 mg/day were 4, 6.5, and 5.7 ng/mL at the aforementioned time points, respectively. Corresponding average anti-Xa levels collected at the same time were 0.07, 0.11, and 0.10 IU/mL, respectively. Average betrixaban concentrations when dosed at 80 mg/day were 10.5, 21.5, and 20.8 ng/mL at the previously mentioned time points, respectively. Corresponding average anti-Xa levels were 0.17, 0.29, and 0.28 IU/mL, respectively [5]. This study indicates that betrixaban has a dose and concentration-dependent impact on anti-Xa levels. It also confirms what was identified in pre-clinical studies, that at therapeutic concentrations of betrixaban, anti-Xa levels are likely to be between 0.09 and 0.44 IU/mL [5].

This is relevant to our patient case because the patient’s anti-Xa levels were between 0.12 and 0.32 IU/mL, which suggests that they were within the expected range reported in prior studies of betrixaban. We did not measure actual betrixaban concentrations, but consistent anti-Xa levels were used as a surrogate pharmacodynamic marker to assess for medication anticoagulant effects and lack of accumulation over time. Based on the EXPERT trial, betrixaban was not yet at steady state on Day 2 of therapy [5]. However, patients in the EXPERT trial were not given a loading dose of betrixaban at initiation of therapy. The study authors suggested that a doubling of the dose on Day 1 would reach 80% of steady state levels on the first day of therapy instead of on Day 3 or 4 [5]. Since our patient was loaded with betrixaban 80 mg on Day 1, it would be expected that levels collected on Day 2 of therapy would be at or near steady-state, and the patient had likely reached full steady-state by Day 7 of therapy.

It is unknown if betrixaban is removed by dialysis. Betrixaban is 60% protein bound, so some removal is possible [3]. Based on the patient’s anti-Xa levels collected before and after dialysis, the anti-Xa levels decreased from 0.19 to 0.12 IU/mL. However, it cannot be confirmed if this was from removal by dialysis or just that it was over 24 hours from the last betrixaban dose, and it was an expected trough concentration, regardless of dialysis. A formalized pharmacokinetic study would be needed to answer that question.

In a post-hoc analysis of the APEX trial, they compared betrixaban full dose (80 mg) and betrixaban reduced dose (40 mg) to prophylaxis doses of enoxaparin [14]. Betrixaban at a reduced dose was used in patients with severe renal dysfunction (CrCl ≥ 15 and <30 ml/min) and patients on concomitant strong P-glycoprotein inhibitors. They found that the full dose of betrixaban was associated with lower rates of VTE and VTE-related events compared to enoxaparin. However, the betrixaban reduced dose was found to be non-inferior to enoxaparin. Median betrixaban concentrations for 80 mg and 40 mg doses were 19 ng/mL and 11 ng/mL, respectively. Although patients with severe renal impairment do not make up a large part of the population on reduced betrixaban dose, it has been theorized that patients with renal impairment may need higher doses of betrixaban [14] [15]. Further studies would be needed in patients with renal dysfunction and patients with ESRD to optimize dosing. However, in the absence of that data, our patient on reduced-dose betrixaban 40 mg daily achieved previously identified therapeutic anti-Xa levels.

Several limitations of our findings in this patient case should be acknowledged. The results and interpreted observations are based on a single patient case and may not be generalizable to other patients. We used anti-Xa measurements as a pharmacodynamic surrogate for pharmacokinetic evaluation in our patient. We could not conduct a thorough pharmacokinetic analysis or measure dialysis clearance of betrixaban, because we were unable to measure actual plasma and dialysis effluent fluid concentrations of betrixaban in our patient. We were unable to conduct outpatient follow-up or additional monitoring after discharge to assess the longer-term safety and efficacy of betrixaban beyond the hospitalization.

## 4. Conclusion

Data assessing betrixaban use in patients with severe renal failure or ESRD is scarce. Given betrixaban’s minimal renal elimination, it would be a reasonable therapeutic alternative for patients who cannot use heparin for VTE prophylaxis. Our patient highlights the successful use of betrixaban for a patient with a history of ESRD. Further studies would be needed to validate use in these patients and evaluate optimal dosing.

## Figures and Tables

**Table 1.** Pre-admission medication list.

Medication	Dose
Carvedilol	12.5 mg Twice Daily
Hydroxychloroquine	200 mg Daily
Lidocaine 5% Patch	1 Patch Daily
Pantoprazole	20 mg Daily
Prednisone	5 mg Daily
Sevelamer	800 mg Three Times Daily
Tiotropium	1 Capsule Daily

**Table 2.** Anticoagulant pharmacodynamic laboratory monitoring results.

	Baseline	14 h Post 80 mg Dose	1 h Post 40 mg Dose	10 h Post 40 mg Dose	17.5 h Post 40 mg Dose (Pre-HD)	25 h Post 40 mg Dose (Post-HD)	Day 7 of Therapy Trough Level	Reference Values
Anti-Xa Level (IU/mL)	Not Assessed	0.18	0.19	0.32	0.19	0.12	0.15	0.09 – 0.44*
INR (IU/mL)	1.1	Not Assessed	1.2	1.3	1.2	1.1	1.1	0.9 – 1.1
PT (sec)	14.3	Not Assessed	15.4	16.0	15.4	14.5	14.4	12.0 – 14.5

* Based on pre-clinical studies, therapeutic concentrations of betrixaban should generate anti-Xa levels between 0.09 and 0.44 IU/mL with our calibrated assay [5].

**Table 3.** Hemoglobin, hematocrit, and platelet laboratory results.

	Baseline	Day 2	Day 3	Day 4	Day 9	Reference Values
Hemoglobin (g/dL)	9.8	10.5	11.9	12.0	11.9	12.1 – 16.3
Hematocrit (%)	32.1	33.0	37.2	37.8	37.4	35.7 – 46.7
Platelets (10^9^/L)	122	115	138	123	177	150 – 400
